# Absorption Loss Modeling Tools for Terahertz Band Drone Communications

**DOI:** 10.3390/s25164957

**Published:** 2025-08-12

**Authors:** Berkay Sekeroglu, Mikail Erdem, Ozgur Gurbuz, Akhtar Saeed, Hayrettin Cagan Sendag, Murat Kulaksizoglu

**Affiliations:** 1 Department of Electrical and Electronics Engineering, Bogazici University, Istanbul 34342, Türkiye; berkay.sekeroglu@std.bogazici.edu.tr; 2 Faculty of Engineering and Natural Sciences, Sabanci University, Istanbul 34956, Türkiye; ozgur.gurbuz@sabanciuniv.edu (O.G.); akhtarsaeed@sabanciuniv.edu (A.S.); hayrettins@sabanciuniv.edu (H.C.S.); mkulaksizoglu@sabanciuniv.edu (M.K.)

**Keywords:** Terahertz communication, ITU, LBLRTM, *am*, HotW, transmittance, molecular absorption, path loss, simulation tools

## Abstract

This paper compares the following four Terahertz (THz) band molecular absorption loss modeling tools: International Telecommunication Union (ITU)-R P.676 model, Line-by-Line Radiative Transfer Model (LBLRTM), Atmospheric Model (*am*), and HITRAN on the Web (HotW). We evaluate the THz band drone communication tools under horizontal and vertical communication scenarios. We use the U.S. Standard 1976 and tropical weather profiles to generate path loss data across different altitudes, frequencies, and distances. We also employ a simple analytical model, fitting the data from the ITU, LBLRTM, and *am* tools to assess its accuracy in predicting path loss. Our results demonstrate high consistency among the tools, with path loss differences becoming more significant in vertical scenarios. This study provides the first comprehensive comparison of four widely used molecular absorption loss modeling tools for THz band drone communications, considering various scenarios and weather conditions.

## 1. Introduction

Today, with the implementation and commercial availability of fifth-generation (5G) technologies, research for higher data rates is shifting toward sixth-generation (6G) and beyond technologies [1,2,3,4,5,6,7], such as extended reality (XR), digital twins, holographic teleportation, wireless AR/VR headsets, and ultra-fast data kiosks, and full immersiveness [2,8], requiring massive data rates and ultra-low latency requirements [2]. To meet these requirements, Terahertz (THz) band communication is considered to be one of the promising enablers offering up to hundreds of Gbps [9] or even a Tbps [10] as these bands are largely unexplored [4,5] and available, while THz frequency bands enable high data rates and offer numerous benefits, propagation at these frequencies is significantly impacted by molecular absorption loss [11,12], and ground-level links are further degraded by line-of-sight blockage from terrain and buildings [13].

To overcome the challenges of the THz band, unmanned aerial vehicles (UAVs) are being integrated into non-terrestrial networks (NTNs) as a complement to terrestrial networks [6,7,14,15], offering flexible capabilities such as remote sensing, portable base stations, and communication infrastructure, in case of emergency [6,7,15,16]. Operating at higher altitudes, UAVs can establish line-of-sight links more easily due to reduced ground-level obstructions and also benefit from relatively lower molecular absorption, due to lower gaseous concentration compared to ground-level links [11]. However, molecular absorption at THz frequencies remains significant even at higher altitudes. Even though molecular absorption loss degrades communication quality even further in the THz band, it enables novel sensing opportunities by using absorption fingerprints of gases, in joint sensing and communication (JSAC) applications [2]. In this study, we focus on low-altitude communication, where drones are considered simple types of UAVs operating at low altitudes, commonly used in many practical short-range applications.

Designing THz band drone communication systems requires accurate modeling of molecular absorption loss with respect to frequency, distance, and altitude, due to the strong molecular absorption characteristics of the THz band. Although current tools can simulate or approximate molecular absorption loss using analytical models or weather profiles, having a simple closed-form model like in [11], dependent on these variables, is advantageous. Additionally, understanding the effects of different frequencies and weather conditions on molecular absorption loss is essential for a comprehensive understanding of the THz band [17], as there are specific transmission windows with low molecular absorption loss that avoid spikes [18]. Moreover, altitude and weather conditions modify air composition and the density of gaseous molecules, while communication distance influences the molecular absorption loss in conjunction with free space spread loss and frequency [3]. In comparison, depending on the specific application, these tools can perform more precise molecular absorption loss calculations using detailed weather profiles. Alternatively, a simplified closed-form analytical model may be utilized for enhanced analytical tractability. Although various molecular absorption loss modeling tools exist, they are not compared comprehensively.

In this paper, considering THz band drone communication scenarios, we evaluate and compare the following four commonly used tools: the ITU-R P.676 (International Telecommunication Union, Geneva, Switzerland) model [17], Line-by-Line Radiative Transfer Model (LBLRTM) (Atmospheric and Environmental Research, Lexington, MA, USA) [19], atmospheric model (*am*) (Harvard–Smithsonian Center for Astrophysics (CFA), Cambridge, MA, USA) [20], and the High-Resolution Transmission molecular-absorption database (HITRAN) [21,22] on the Web (HotW) (Harvard–Smithsonian Center for Astrophysics (CFA), Cambridge, MA, USA) [23]. In our earlier work [3], our preliminary results are presented for three of these tools (ITU, LBLRTM, *am*) under a horizontal drone-to-drone scenario and a tropical weather profile. In addition to the previous study [3], this paper includes the U.S. Standard 1976 profile [24] in the comparison, along with vertical drone-to-ground and drone-to-drone scenarios, exploring and comparing the fit of the simple analytical model from [11] to the data generated by the tools. To the best of our knowledge, this paper is the first study that compares these tools in such detail, particularly in drone-based applications. It is concluded that similar communication scenarios and weather settings can be simulated with these tools, and the proposed analytical model in [11] can be fitted to the data with low error.

## 2. THz Band Attenuation

In this section, we briefly explain the concepts used in the paper, such as transmittance and path loss. Then, we shortly explain the simple analytical model [11], which is employed to obtain the path loss analytically, and the data generated by tools is used in this model for another comparison.

### 2.1. Transmittance and Path Loss

Transmittance ( τ ) represents the proportion of the received electromagnetic (EM) power ( $P_{\mathrm{RX}}$ ) to the transmitted EM power ( $P_{\mathrm{TX}}$ ) through the medium, with values ranging from 0 to 1 [3]. Transmittance can be modeled as being dependent on the frequency (*f*) of the EM wave, the distance (*d*) of the communication link, the ambient temperature (*T*), the atmospheric pressure ( ρ ), and the water vapor concentration (*V*) [25]. In an aerial communication scenario and under specific weather conditions, it is practical to model the molecular absorption loss with respect to the altitudes of the transmitter ( $h_{1}$ ) and the receiver ( $h_{2}$ ) as follows [26]:

(1) $$\tau \left(d,f,T\left(h_{1},h_{2}\right),\rho \left(h_{1},h_{2}\right),V\left(h_{1},h_{2}\right)\right)=\frac{P_{\mathrm{RX}}}{P_{\mathrm{TX}}}.$$

Since the ambient temperature (*T*), atmospheric pressure ( ρ ), and water vapor concentration (*V*) all depend on $h_{1}$ and $h_{2}$ , which characterize the medium of the communication link under a specific weather setting [25], transmittance can be modeled with respect to $h_{1}$ and $h_{2}$ . Moreover, this altitude-dependent modeling can be achieved by considering specific weather profiles, such as a tropical weather profile or the U.S. Standard 1976 weather profile [24].

Either transmittance ( τ ) or molecular absorption loss ( $A_{\mathrm{abs}}$ ) is modeled in any THz band attenuation model. Molecular absorption loss, the multiplicative inverse of transmittance, represents the attenuation caused by atmospheric gases absorbing the emitted wave [3]. Molecular absorption loss ( $A_{\mathrm{abs}}$ ) in dB scale can be formulated as [3]:

(2) $$A_{\mathrm{abs}}\left(d,f,h_{1},h_{2}\right)\left[\mathrm{dB}\right]=10\log_{10}\left(\frac{1}{\tau (d,f,h_{1},h_{2})}\right).$$

In addition to the molecular absorption loss, free space path loss (FSPL) also contributes to the reduction of signal power as the signal spreads through the space, and FSPL, being dependent on the distance of the communication link and the frequency of the wave, is calculated as follows [27]:

(3) $$A_{\mathrm{fspl}}\left(d,f\right)\;\left[\mathrm{dB}\right]=20\log_{10}\left(\frac{4\pi df}{c}\right),$$

where *c* represents the speed of the THz wave in free space, which is equal to 299,792,458 (m/s).

Total path loss ( $A_{\mathrm{PL}}$ ) can be calculated as the sum of the molecular absorption loss ( $A_{\mathrm{abs}}$ ) and FSPL ( $A_{\mathrm{fspl}})$ in dB scale. Modeling the total path loss with respect to the distance of the communication link (*d*), the frequency of the wave (*f*), and the altitudes of the transmitter ( $h_{1}$ ) and the receiver ( $h_{2}$ ), total path loss ( $A_{\mathrm{PL}}$ ) can be obtained as follows [27]:

(4) $$A_{\mathrm{PL}}\left(d,f,h_{1},h_{2}\right)\;\left[\mathrm{dB}\right]=A_{\mathrm{abs}}\left(d,f,h_{1},h_{2}\right)\;\left[\mathrm{dB}\right]+A_{\mathrm{fspl}}\left(d,f\right)\;\left[\mathrm{dB}\right].$$

**Figure 1:**
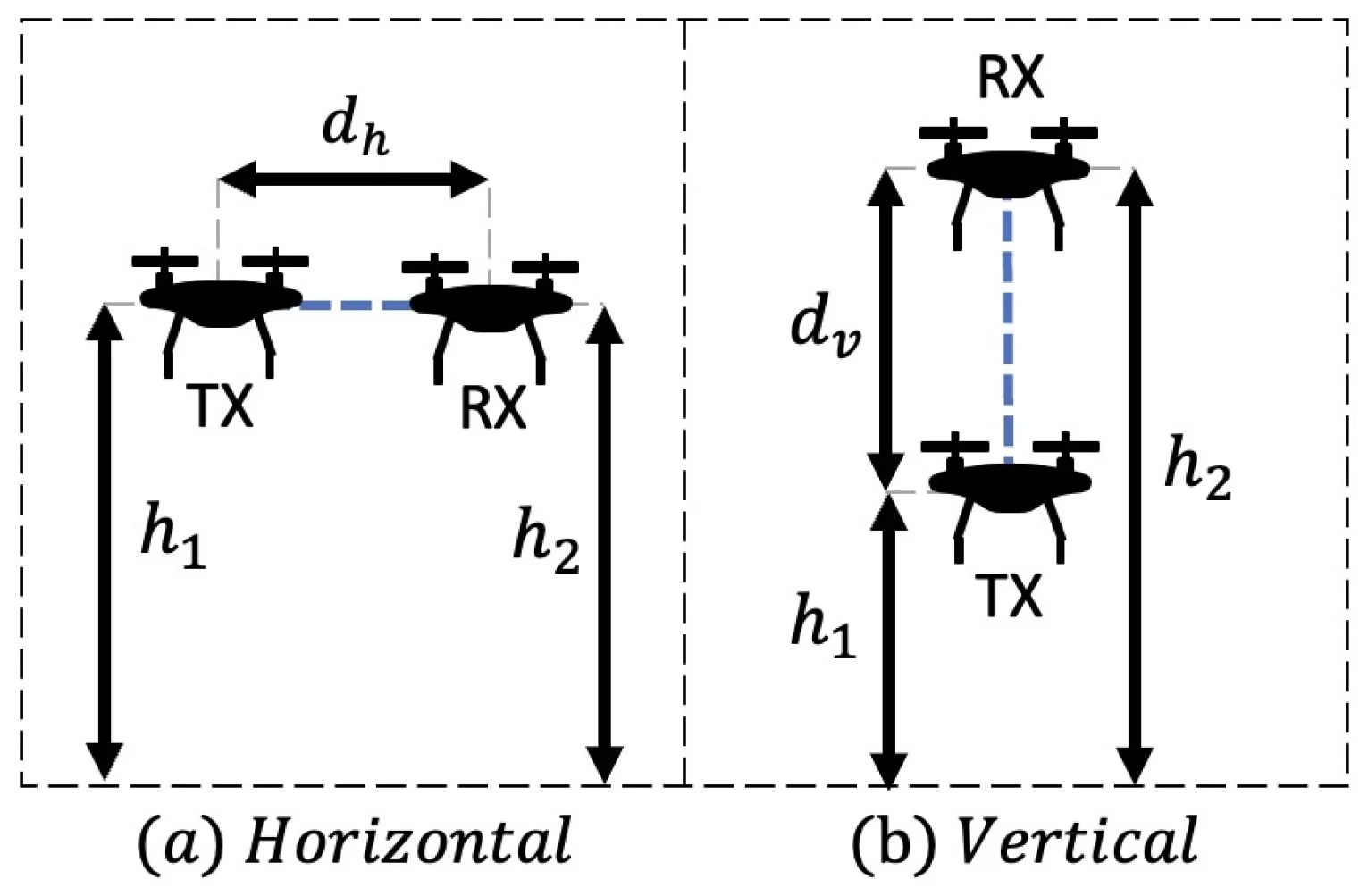
Drones under horizontal and vertical communication scenarios.

The distance (*d*) between the drones and the altitudes ( $h_{1},h_{2}$ ) of the drones fully characterize the positioning of the drones. For a given frequency (*f*) of communication, and given the type of scenario (horizontal or vertical), the distance (*d*) and the altitude of one of the drones (e.g., $h_{1}=h$ ) are sufficient to deduce $h_{2}$ ; hence path loss can be modeled as $A_{\mathrm{PL}}\left(d,f,h\right)$ . As shown in root mean square error (NRMSE) Figure 1, under a horizontal communication scenario *h*, denoting the altitude of the drones and the distance between them $d_{h}$ , would be sufficient to model the path loss. For a vertical scenario, the altitude of the transmitter ( $h_{1}=h$ ) and the distance between drones ( $d_{v}$ ) would be adequate.

### 2.2. A Simple Analytical Model

The simple analytical model proposed in [11], a frequency, distance, and altitude-dependent model for the THz band, is employed to compare the performance of the molecular absorption loss modeling tools in Section 4. In [11], a water-filling capacity-maximisation algorithm over the common 0.75–1 THz range (where all three tools commonly operate) identified two optimum windows of frequencies: 0.79–0.91 THz (Band 1) and 0.93–0.94 THz (Band 2). The coefficients of this model are determined through a cascaded regression of transmittance data obtained from the LBLRTM for horizontal communication. In the original study [11], altitudes from sea-level to 1 km and distances from 1 to 100 m are considered.

The simple analytical model ( $\hat{\tau }$ ) for approximating transmittance ( τ ) is given as follows [11]:

(5) $$\hat{\tau }\left(d,f,h\right)=C_{1}e^{\left(\sum_{p=0}^{P}{\hat{\lambda }}_{p}f^{p}\right)e^{C_{2}h}d},$$

where the model is the cascade modeling of transmittance with respect to distance (*d*), altitude (*h*), and frequency (*f*). The cascade modeling of exponential *P* is the order of the polynomial model, accounting for modeling frequency. The optimal value for *P*, the order of the polynomial, was found as 8 for Band 1 and 4 for Band 2, for LBLRTM data, under a horizontal scenario and a tropical weather profile. The same hyperparameter values for the model are used throughout this study. The details of the optimization of coefficients (the coefficients of the polynomial and constants $C_{1}$ and $C_{2}$ ) can be found in [11].

## 3. THz Band Absorption Loss Modeling Tools

This section summarizes the four THz band transmittance tools. There exist some other commercial THz band molecular absorption loss modeling tools, like MODerate resolution atmospheric TRANsmission (MODTRAN) (Spectral Sciences Inc. (SSI), Burlington, MA, USA; and Air Force Research Laboratory (AFRL), Wright–Patterson Air Force Base, OH, USA) [28]. However, we opt not to include such commercial tools. Furthermore, MODTRAN announces that its line-by-line transmittance results are similar to LBLRTM [28]. Specifically, International Telecommunication Union (ITU) P.676 model [17], Line-by-Line Radiative Transfer Model (LBLRTM) [19], atmospheric model (*am*) [20], and HITRAN on the Web (HotW) [23]. In this study, LBLRTM version 12.2 is used.

The altitude range for all tools depends on the availability of the corresponding weather data, whether predefined or user-defined, while the tools have no specific altitude limits, some may have limits based on the available predefined weather profiles. Moreover, in this section, the tropical and U.S. Standard 1976 weather profiles are briefly described.

Depending on the tool, the output may be provided as either transmittance or molecular absorption loss. To enable a consistent comparison across tools, we convert between these representations when necessary, and thus use both terms interchangeably throughout the manuscript, while favoring ’molecular absorption loss’ unless transmittance is the native output.

### 3.1. ITU Model

The ’Recommendation ITU-R P.676’ model is a widely adopted recommendation by ITU, to estimate the attenuation due to atmospheric gases, such as oxygen and water vapor, for terrestrial and slant paths [17]. The model is valid over a frequency range of 1–1000 GHz. The attenuation is accurately calculated by a line-by-line calculation method for up to 1000 GHz. The recommendation also provides a simplified method to estimate the attenuation for frequencies up to 350 GHz. This model uses signal path length, carrier frequency, ambient temperature, atmospheric pressure, and atmospheric water vapor density, and calculates the signal attenuation.

In this study, the MATLAB R2024a (The MathWorks, Natick, MA, USA) function ‘gaspl’ is used to calculate the attenuation of atmospheric gases [29] since the ITU-R P.676 model [17] can be easily accessed as a built-in function in MATLAB Radar Toolbox. Additionally, the ITU-Rpy library in Python 3.9 [30] provides access to this model. The ITU-Rpy library includes the newer versions of the model, as well. This library also offers access to other ITU recommendations, such as ITU-R P.835 [31], which provides equations to calculate temperature, pressure, and water vapor content based on the U.S. Standard Atmosphere 1976 model [24]. For the U.S. Standard 1976 weather profile [24], Recommendation ITU-R P.835-6 [31] can be used with the ’ atmositu’ function [32] in MATLAB, as explained in Section 3.5.

### 3.2. LBLRTM Tool

Line-by-Line Radiative Transfer Model (LBLRTM) is an advanced and highly accurate software package used for calculating the radiative transfer of atmospheric gases, developed by Atmospheric and Environmental Research (AER) [33]. It has been validated for accuracy using atmospheric radiance spectra across various wavelengths, from ultraviolet to sub-millimeter. LBLRTM uses the HITRAN database to obtain a line database for line parameters. It can also account for additional absorption by water vapor, carbon dioxide, methane, oxygen, and nitrogen by utilizing spectroscopic databases [3,33]. LBLRTM offers compatibility with other radiative transfer tools. It is designed to be computationally efficient while being accurate, making it practical to perform detailed line-by-line calculations for large datasets.

LBLRTM is designed to work for the frequency range of 0.75–10 THz. LBLRTM has 6 predefined weather profiles, as follows: tropical, mid-latitude summer, mid-latitude winter, sub-arctic summer, sub-arctic winter, and the U.S Standard 1976. Additionally, the user can supply a different atmospheric profile. The inputs LBLRTM requires are the positions of the transmitter and the receiver, the distance of the link, and the weather profile. With these inputs, LBLRTM computes the atmospheric transmittance for a given range of wavenumbers. The installation process for this tool is more complex, and its operation requires a higher level of expertise compared to the other transmittance tools.

### 3.3. am Tool

The “atmospheric model” (*am*) [20], is a detailed and flexible tool developed by Harvard-Smithsonian Center for Astrophysics (CFA), for radiative transfer, optical depth, and refraction computations across microwave to sub-millimeter wavelengths, offering line-by-line calculations. It supports various molecular species (column types) and isotopes, using data from the HITRAN database [22,34]. Designed to model atmospheric propagation, it handles diverse scenarios, from radio astronomy and laboratory testing to any problem that can be modeled as a narrow beam propagating through user-defined path segments. The segments of the propagation path in *am* are represented by layers, and each layer is a mixture of the absorbing species (namely, column types) between the boundaries of that layer. The type and density of species within a layer (referred to as columns) are individually defined [34].

By specifying parameters such as the frequency resolution range, background temperature, pressure, layer thickness, and volume mixing ratio, the *am* tool can accurately calculate transmittance values. These parameters define the properties of each layer within the model, allowing the tool to compute essential spectral characteristics, such as opacity, transmittance, and other radiative properties. Depending on the weather profile being used, the volume mixing ratio can be determined from the altitude of communication as described in Section 3.5. *am* tool is designed to work for a frequency range of 0–15 THz. The *am* tool is highly advanced and powerful; with a very detailed and well-structured manual and documentation, the software is easy to install and use. Although unexpected problems and errors might not be directly covered, a solid knowledge base of the manual and the software combined with the available documentation would be helpful to resolve most issues effectively.

### 3.4. HotW Tool

HITRAN on the Web (HotW) [23] is an online platform that provides access to information on the comprehensive HITRAN database [22], which contains spectral line parameters of atmospheric molecules and pollutants. The tool is essential for tasks related to atmospheric optics and molecular absorption spectra modeling, allowing for the calculation of absorption and transmission functions for predefined gas mixtures. These gas mixtures include air compositions of pure molecules and the USA models for different latitudes.

HotW covers the infrared region of the spectrum, but it has been extended to include ranges from microwave to ultraviolet. HotW allows users to analyze spectroscopic data from the HITRAN database by specifying parameters such as molecular species, temperature and pressure, and spectral line shapes. It also supports detailed modeling of high-resolution spectra, allowing for the simulation of spectra for various gas mixtures and a range of wavenumbers [23]. It provides a user-friendly web-based interface for researchers to access the HITRAN database easily.

Table 1 provides a summary of THz band modeling tools. By providing appropriate input, accurate output can be obtained as either transmittance or molecular absorption loss. The table also shows boundaries as frequency range and resolution. Installation and documentation are also important criteria in tool selection. In the next section, the tools are compared in detail in terms of transmittance, hence calculated path loss values for different communication scenarios and weather profiles, as well as varying altitudes and distances. Before path loss simulations, the two weather profiles and how they are considered in the tools are described in the following subsection.

### 3.5. Weather Profiles

The molecular absorption loss modeling tools use data regarding the weather composition, temperature, pressure, humidity, or even the concentration of other types of molecules to accurately simulate how radiation is absorbed, emitted, and transmitted. Here, a tropical weather profile is used for comparison because it has the highest water vapor concentration and the highest molecular absorption loss. Moreover, the U.S. Standard 1976 weather profile is also used since it is counted as standard, and the same input is expected to generate the same output in all tools [24]. We interpolate the available weather data to estimate values at altitudes (*h*) between the provided data samples.

**Table 1:** A summary of THz band absorption modeling tools.

	ITU Model	LBLRTM Tool	*am* Tool	HotW Tool
**Input**	Distance, Frequency, Temperature, Pressure, Water Vapor Density	Distance, Altitudes *, Zenith Angle, Weather Profile, Wavenumbers	Distance, Frequency, Temperature, Pressure, Volume Mixing Ratio	Distance, Temperature, Pressure, Weather Profile, Wavenumbers
**Output**	Absorption Loss in dB	Transmittance	Transmittance	Transmittance
**Frequency range**	1 GHz–1 THz	0.75 –10 THz	0–15 THz	Depends on the species in the gas mixture.
**Frequency resolution**	<0.01 KHz	0.3 GHz	<0.01 KHz	∼0.2997 MHz
**Installation**	No need for installation (Works via a built-in function in MATLAB and a library in Python.)	Very Difficult	Easy	No need for installation (Works on the website.)
**Documentation**	Very Good	Good	Very Good	Good

* All tools are capable of calculating molecular absorption loss at different altitudes when they are provided with the weather profile data for each altitude. The LBLRTM tool involves predefined weather profiles that take into account the altitude information, so it directly enables simulations at given input altitudes. For the ITU model and the *am* tool, data (temperature, pressure, water vapor density, etc.) for considered altitudes need to be provided manually, since these tools do not have predefined weather profiles. The HotW tool provides weather data for the considered profiles, but only at sea-level; hence, simulations with HotW are limited to sea-level conditions.

For the tropical weather profile, in the *am* tool, we have used the annual tropical weather layers provided in the “*am* cookbook” [20], under the “zonal” folder, which is documented as being derived from the National Aeronautics and Space Administration (NASA) Modern-Era Retrospective analysis for Research and Applications, Version 2 (MERRA-2) reanalysis [35]. LBLRTM has a tropical weather profile provided for use. For the ITU model, we have used the same data from the “*am* cookbook” [20]. For HotW, we have used the predefined gas mixture, “USA model, tropics, H=0”, defined only for the sea-level. Table 2 lists the temperature, pressure, and water vapor content of the weather for the tropical weather profile at six altitudes (*h*), as follows: 110, 330, 560, 790, 1020, and 1260 m. The provided tropical weather profile data are from the “*am* cookbook” [20].

For the U.S. Standard 1976 weather profile [24], the temperature and pressure data are obtained from the “atmositu” function in MATLAB, which provides an approximation of the U.S. Standard Atmosphere 1976 with insignificant relative error [32]. The function gives outputs as absolute temperature, atmospheric pressure, and water vapor density for an altitude above sea-level, desired latitude interval, and season (either summer or winter). The water concentration data for the U.S. Standard 1976 weather profile is documented in [36,37]. Table 3 lists the temperature, pressure, and water vapor content of the weather for the U.S. Standard 1976 weather profile [24], from [36,37] at the following three altitudes (*h*): 0, 1000, and 2000 m.

## 4. Evaluations via Path Loss Simulations

This section assesses the molecular absorption loss modeling tools through simulations for THz band drone communication. For the comparison of tools, we first provide a comparison of four tools (ITU, LBLRTM, *am*, and HotW) under a horizontal communication scenario, at the sea-level, for the U.S. Standard 1976 weather profile [24] and a tropical weather profile. Then, ITU, LBLRTM, and *am* are compared for various altitudes for horizontal and vertical scenarios under two weather profiles. Moreover, the simple analytical model [11] is employed to fit the data from these tools, and the normalized root mean square error results obtained from each tool are compared.

For the analytical model, we use the same model with the same hyperparameters (an exponential model with a polynomial order of 8 for Band 1 and a polynomial order of 4 for Band 2) for all tools and scenarios and fit the coefficients of the simple analytical model to the data obtained from a tool for each scenario and weather profile separately. Thus, although the model form and hyperparameters are fixed, the regression coefficients are independently optimized for each tool and scenario. Accordingly, the reported NRMSE values reflect the quality of fit for each specific tool and scenario combination. Note that we exclude HotW from analyses in the last two subsections, as it enables simulations only at the sea-level for the considered weather profiles.

### 4.1. Sea-Level Simulations

For the sea-level simulations, two nodes at an altitude of 0 m ( $h_{1}=h_{2}=0$ m) are considered, separated by a distance ( $d_{h}$ ) of 1 m. Given that predefined weather profiles are only provided at the sea-level, we have decided to use the “USA model, tropics, H = 0” for the tropical weather profile and the “USA model, high latitude, summer, H = 0” for the U.S. Standard 1976 profile, for comparison, as they provide the closest levels of gas mixtures available on HotW.

**Figure 2:**
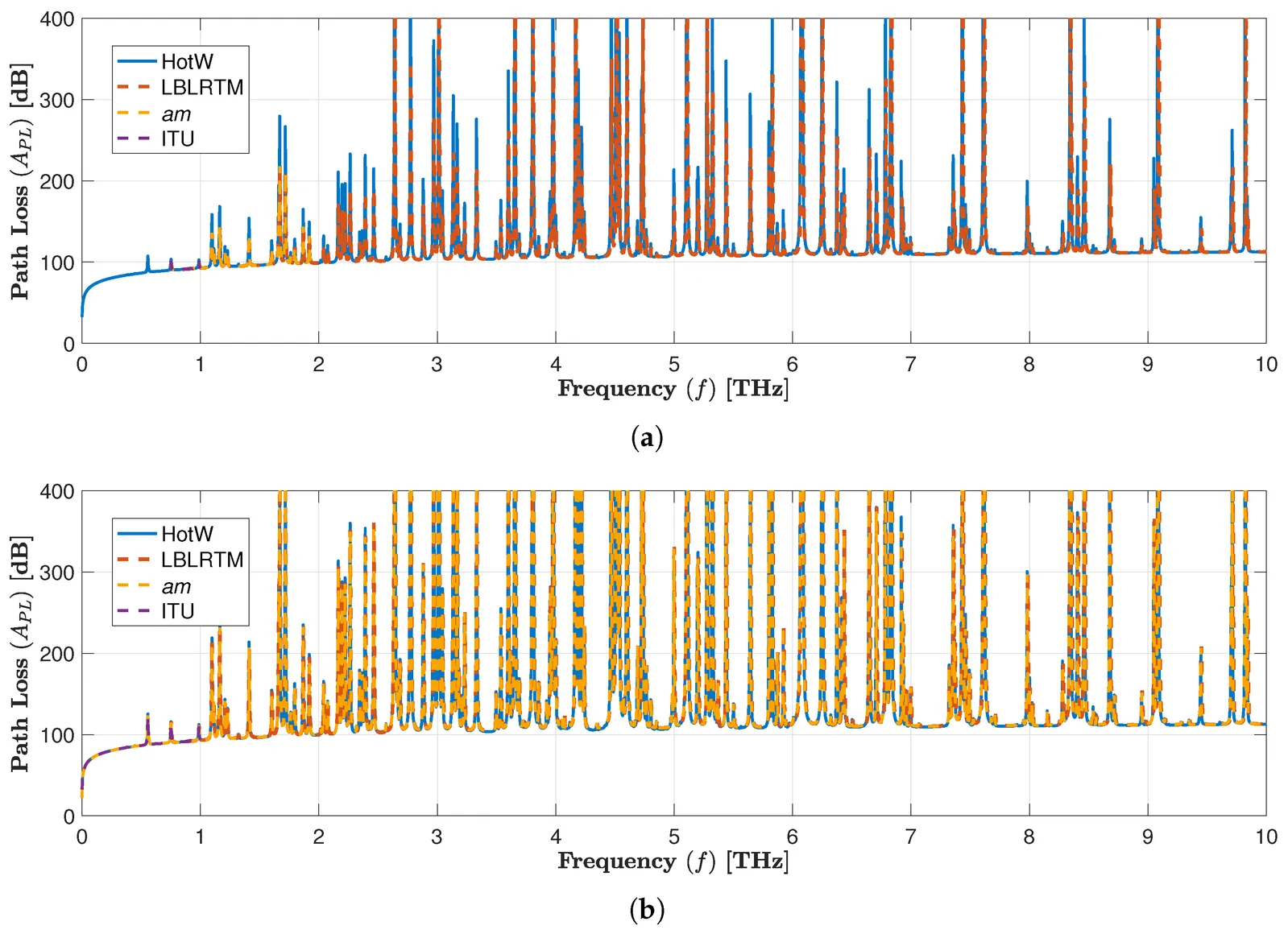
Path Loss with respect to frequency for drones at sea-level ( h=0 m), and $d_{h}=1$ m apart. (**a**) ‘USA model, high latitude, summer’ for HotW, tropical weather profile for LBLRTM, am , and ITU. (**b**) ‘USA model, tropics’ for HotW, the U.S. Standard 1976 weather profile for LBLRTM, am , and ITU.

Figure 2 shows the path loss data generated using HotW, LBLRTM, *am*, and ITU, for horizontal communication at the sea-level and for drones that are 1 m apart considering tropical and the U.S. Standard 1976 weather profiles. Once the transmittance or molecular absorption loss is calculated using the tools, the path loss is determined using (4) by adding the FSPL to the molecular absorption loss, as explained in Section 2.1. The figure shows that the HotW model exhibits minor deviations from the other tools. This is due to differences in how weather profiles are specified across tools. The inputs of the three tools (ITU, *am*, and LBLRTM) are matched to represent the same weather profile as closely as possible. For HotW, the selected profiles are the ‘USA model, high latitude, summer’ and ‘USA model, tropics’, since these profiles are the closest available equivalents to the profiles used in the other tools. It can be inferred that none of the tools significantly differ from the others as far as the sea-level communication considered. Since the pre-defined weather profiles (USA Model Tropics and USA Model High Latitude Summer) for HotW are only available for the sea-level, we consider HotW only for the sea-level scenario, and not for the horizontal and vertical scenarios.

### 4.2. Horizontal Drone Scenarios

In this subsection, we explore the modeling tools under horizontal communication scenarios, considering various realistic drone altitudes, where the transmitter and receiver drones are at the same altitude ( $h_{1}=h_{2}=h$ ), and the distance between the transmitter and the receiver is depicted with $d_{h}$ , as shown in Figure 1a. Moreover, we also consider the simple analytical model [11], concerning distance ( $d_{h}$ ), frequency (*f*), and altitude (*h*).

Since drone communication is considered, altitudes ranging from 0 to 500 m are analyzed for path loss with a step increase of 10 m. The distance between the drones varies from 1 to 100 m, incrementing by 1 m. Note that in the figures, we present results for the altitudes of 10, 100, and 500 m, and the distances of 10, 50, and 100 m whereas all considered altitude and distance values are taken into consideration for overall error calculations of the tools. After calculating the transmittance or absorption from the tools, we calculate the path loss using (4) by adding the FSPL as described in Section 2.1.

**Figure 3:**
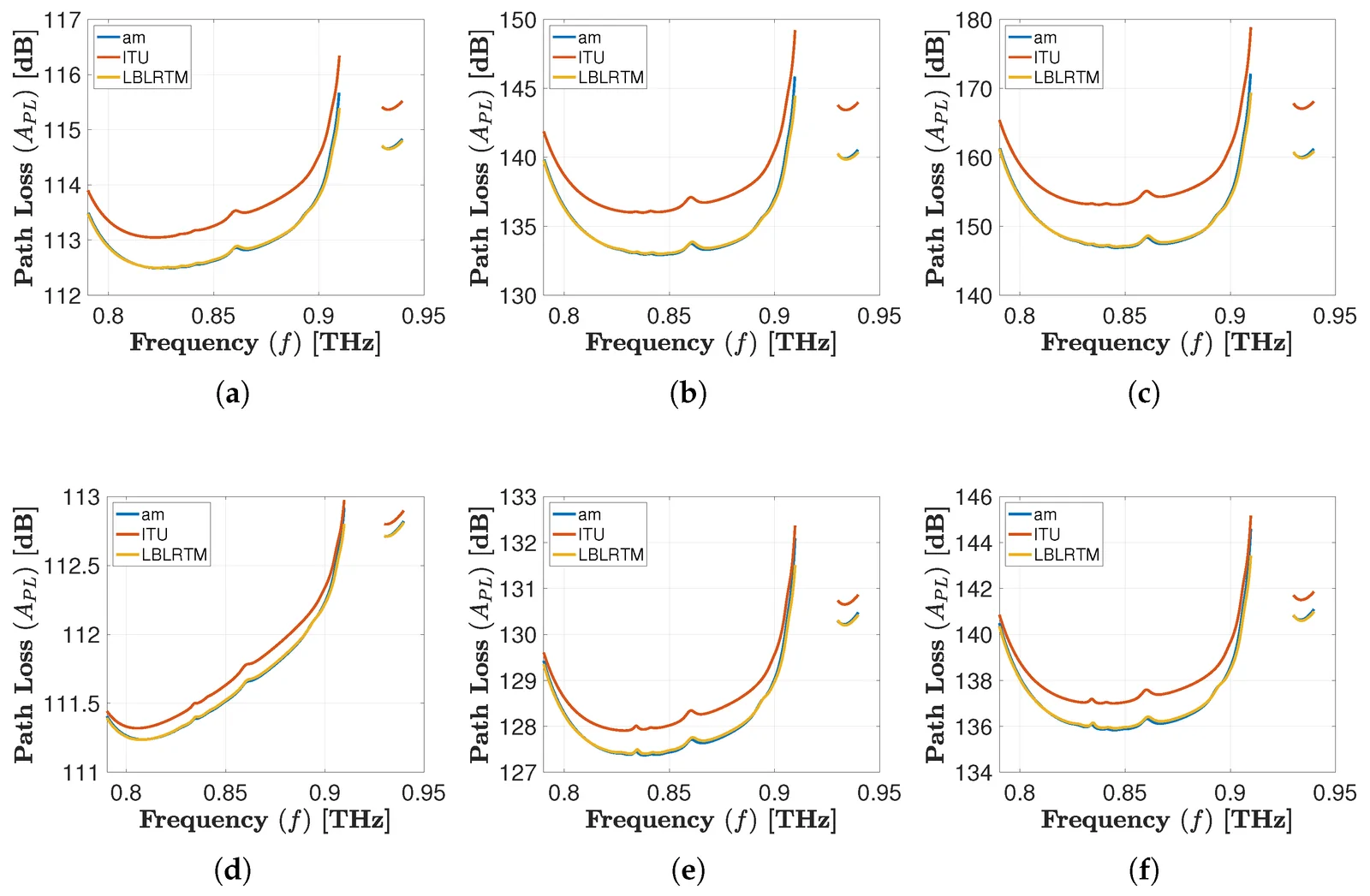
Path Loss in dB, for all three tools under a Tropical weather profile (top row) and U.S. Standard 1976 weather profile (bottom row), for horizontal communication at various distances and h=100 m, drones being $d_{h}$ meters apart horizontally (Only data points inside Band 1 (0.79–0.91 THz) and Band 2 (0.93–0.94 THz) are plotted, appearing discrete). (**a**) Tropical, at $d_{h}=10$ m. (**b**) Tropical, at $d_{h}=50$ m. (**c**) Tropical, at $d_{h}=100$ m. (**d**) U.S. Standard 1976, at $d_{h}=10$ m. (**e**) U.S. Standard 1976, at $d_{h}=50$ m. (**f**) U.S. Standard 1976, at $d_{h}=100$ m.

Figure 3 depicts total path loss with respect to the frequency at h=100 m altitude under a tropical weather profile and the U.S. Standard 1976 profile. In Figure 3a–c, path loss values for the tools under the tropical weather profile, for three different distances $d_{h}=10,50,100$ m are given. In the next row, in Figure 3d–f, path loss values for the tools under the U.S. Standard 1976 profile at three different distances are given. As expected, the path loss for the tropical weather profile is higher, due to the increased water vapor concentration. As seen in the figure, the difference between *am* and LBLRTM is negligible, while ITU has a slight difference.

Fitting of the simple analytical model to the data are carried out for three tools (LBLRTM, ITU, and *am*) and for two weather profiles (tropical and U.S. Standard 1976), separately. The choice of frequency sub-bands in this study follows the rationale established in [38], where these bands were selected because the resource allocation methods discussed in [38] identified them as those containing the highest capacity channels within the 0.75 –10 THz frequency range. Since the 0.75–1 THz range is the only frequency interval common to all three tools (LBLRTM: 0.75 –10 THz; ITU: 1 GHz–1 THz), our earlier work [11] confined the analysis to this range. In that study, we identified two favorable windows, 0.79–0.91 THz (Band 1) and 0.93–0.94 THz (Band 2), using a water-filling optimization that maximizes channel capacity while minimizing atmospheric attenuation for drone links. We therefore adopt the same sub-bands here to enable a consistent comparison of the absorption loss modeling tools.

**Figure 4:**
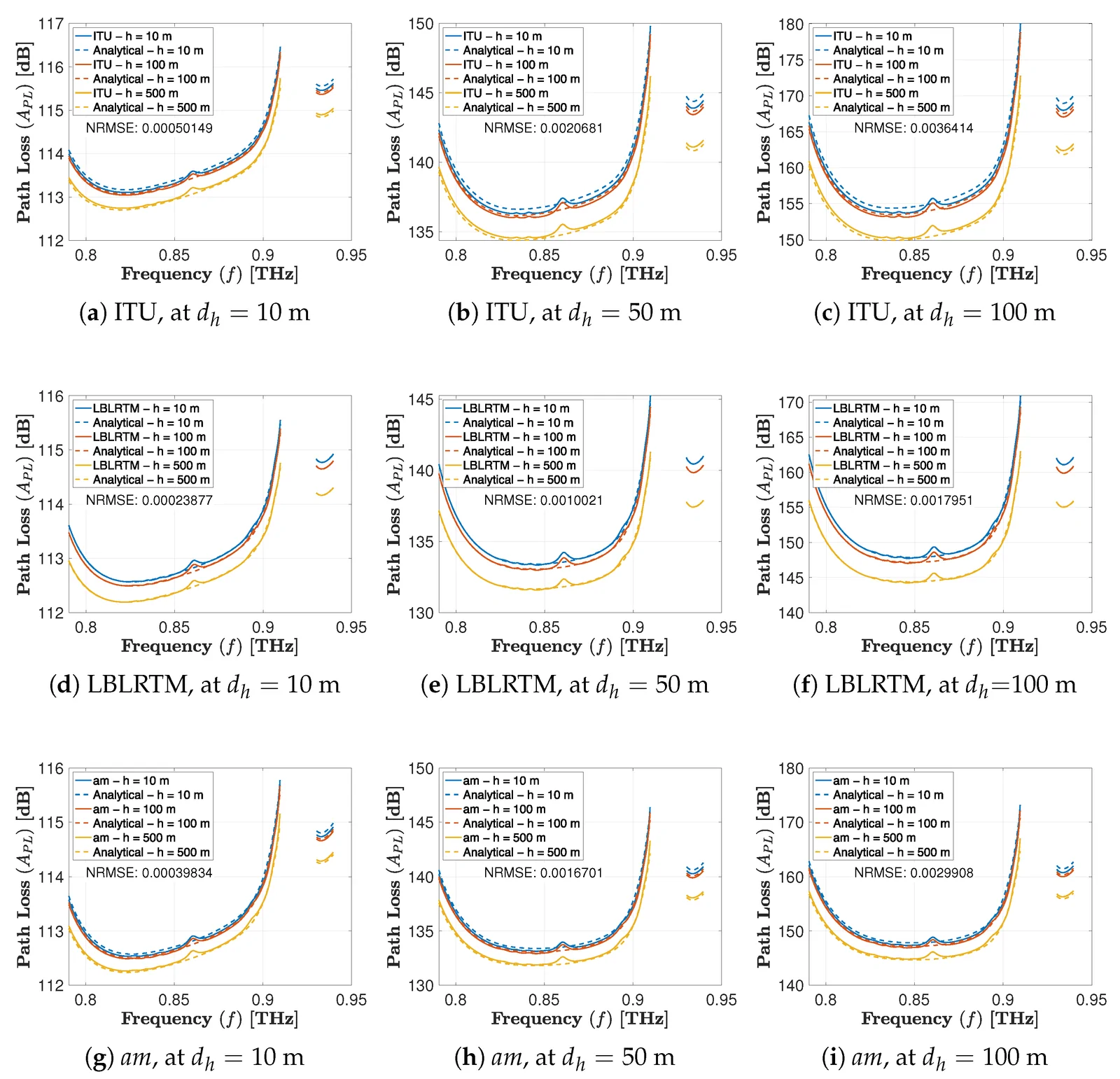
Path Loss in dB and the simple analytical model (dashed lines) [11] for ITU, LBLRTM and *am* under a tropical weather profile, for horizontal communication at various distances and h=10,100,500 m, drones being $d_{h}$ meters apart horizontally (Only data points inside Band 1 (0.79–0.91 THz) and Band 2 (0.93–0.94 THz) are plotted, appearing discrete).

In Figure 4, total path loss and the simple analytical model [11] with respect to frequency for horizontal communication under a tropical weather profile are plotted. In each subfigure, the simple analytical model is fitted to data generated by that tool and plotted for three different altitudes ( h=10,100,500 m). For each tool (given in a row), three distances are considered, $d_{h}=10,50,100$ m). In each subfigure, the normalized root mean square error (NRMSE) values are shown, which are calculated as the error between the data from the tool and the simple analytical model fitted for that tool, for the data points in the figure. We quantify the fit between the tool’s path-loss data and our analytical model via the root-mean-square error (RMSE) in (6), and then divide by the mean path loss to obtain the normalized RMSE (NRMSE) in (7).

(6) $$\mathrm{RMSE}=\sqrt{\frac{1}{N}\sum_{i=1}^{N_{d}}\sum_{j=1}^{N_{f}}\sum_{k=1}^{N_{h}}{\left(\begin{array}{c} {\hat{A}}_{PL}\left(d_{i},f_{j},h_{k}\right)-\\ \;\;\;A_{\mathrm{PL}}\left(d_{i},f_{j},h_{k}\right) \end{array}\right)}^{2}},$$

(7) $$\mathrm{NRMSE}=\frac{\mathrm{RMSE}}{{\mathrm{mean}}_{\begin{array}{c} i=1,2,\ldots ,N_{d}\\ j=1,2,\ldots ,N_{f}\\ k=1,2,\ldots ,N_{k} \end{array}}\left(A_{\mathrm{PL}}\left(d_{i},f_{j},h_{k}\right)\right)},$$

where $A_{\mathrm{PL}}\left(d,f,h\right)$ is the total path loss data calculated with the tool, and the total path loss modeled by the fitted simple analytical model is denoted as ${\hat{A}}_{PL}\left(d,f,h\right)=10\log_{10}\left(1/\hat{\tau }\left(d,f,h\right)\right)+20\log_{10}\left(\{4\pi df\}/c\right)$ , as given in Section 2.2. In (7), $d_{i}$ is the *i*’th distance sample ( $d_{i}=1,2,\ldots N_{d}$ ), $f_{j}$ is the *j*’th frequency sample ( $j=1,2,\ldots N_{f}$ ), and $h_{k}$ is the *k*’th altitude sample ( $k=1,2,\ldots N_{h}$ ). Having $N_{d}\times N_{f}\times N_{h}$ samples in total, normalizing RMSE with the mean value of data, gives NRMSE.

**Figure 5:**
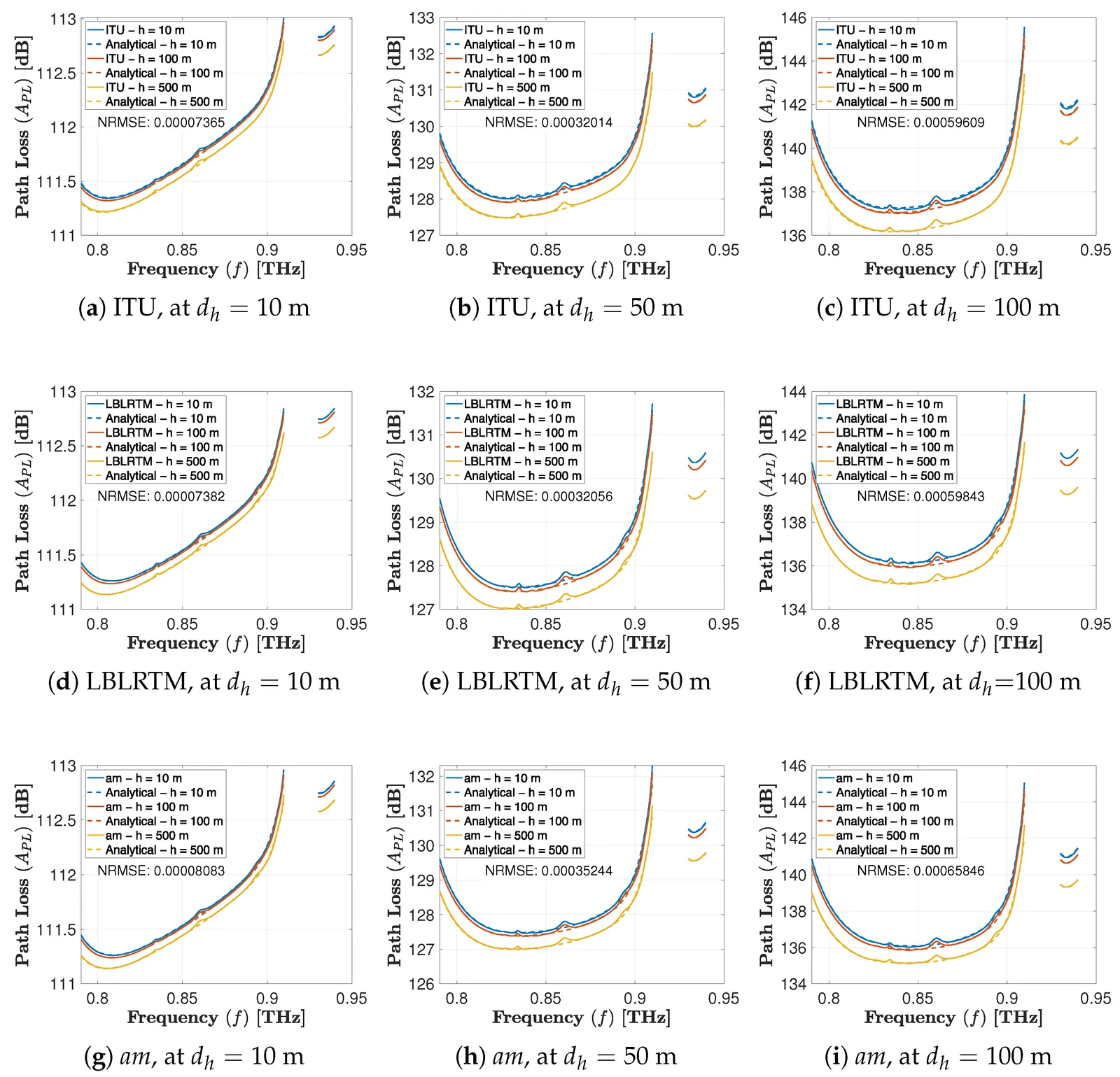
Path Loss in dB and the simple analytical model (dashed lines) [11] for ITU, LBLRTM and *am* under the U.S. Standard 1976 profile, for horizontal communication at various distances and h=10,100,500 m, drones being $d_{h}$ meters apart horizontally (Only data points inside Band 1 (0.79–0.91 THz) and Band 2 (0.93–0.94 THz) are plotted, appearing discrete).

Similarly, Figure 5 illustrates the results of the simple analytical model and the tools for horizontal communication under a U.S. Standard weather profile. NRMSE values for the subfigures are presented in the same way. It is to be noted that although the simple analytical model in [11] was initially designed for LBLRTM, it achieves a sufficiently close fit across all tools, depicting the consistency of the data and robustness of the analytical model.

### 4.3. Vertical Drone Scenarios

As illustrated in Figure 1, drones are positioned vertically up and down for the vertical scenarios, considering drone-to-ground and drone-to-drone communication scenarios. The same three tools (LBLRTM, ITU, *am*) are considered under two weather profiles in these tests. We generate the data and model the total path loss using the same simple analytical model [11], with respect to distance ( $d_{v}$ ), frequency (*f*), and altitude (*h*). The transmitter and receiver are aligned, with one at a height of $h_{1}=h$ m and the other at a height of $h_{2}=h+d_{v}$ m, to establish direct vertical communication. The distance between the transmitter and the receiver is $d_{v}$ , the subscript denoting the vertical communication scenario.

Moreover, in the vertical scenario, altitudes ranging from 0 to 500 m in steps of 10 m and distances between drones ranging from 1 to 100 m in steps of 1 m are considered. Note that in the figures, we present results for the altitudes of 10, 100, and 500 m, and the distances of 10, 50, and 100 m whereas all considered altitude and distance values are taken into consideration for overall error calculations of the tools. After generating the transmittance or molecular absorption loss data using the tools, we calculate the total path loss by adding the FSPL as described in Section 2.1.

**Figure 6:**
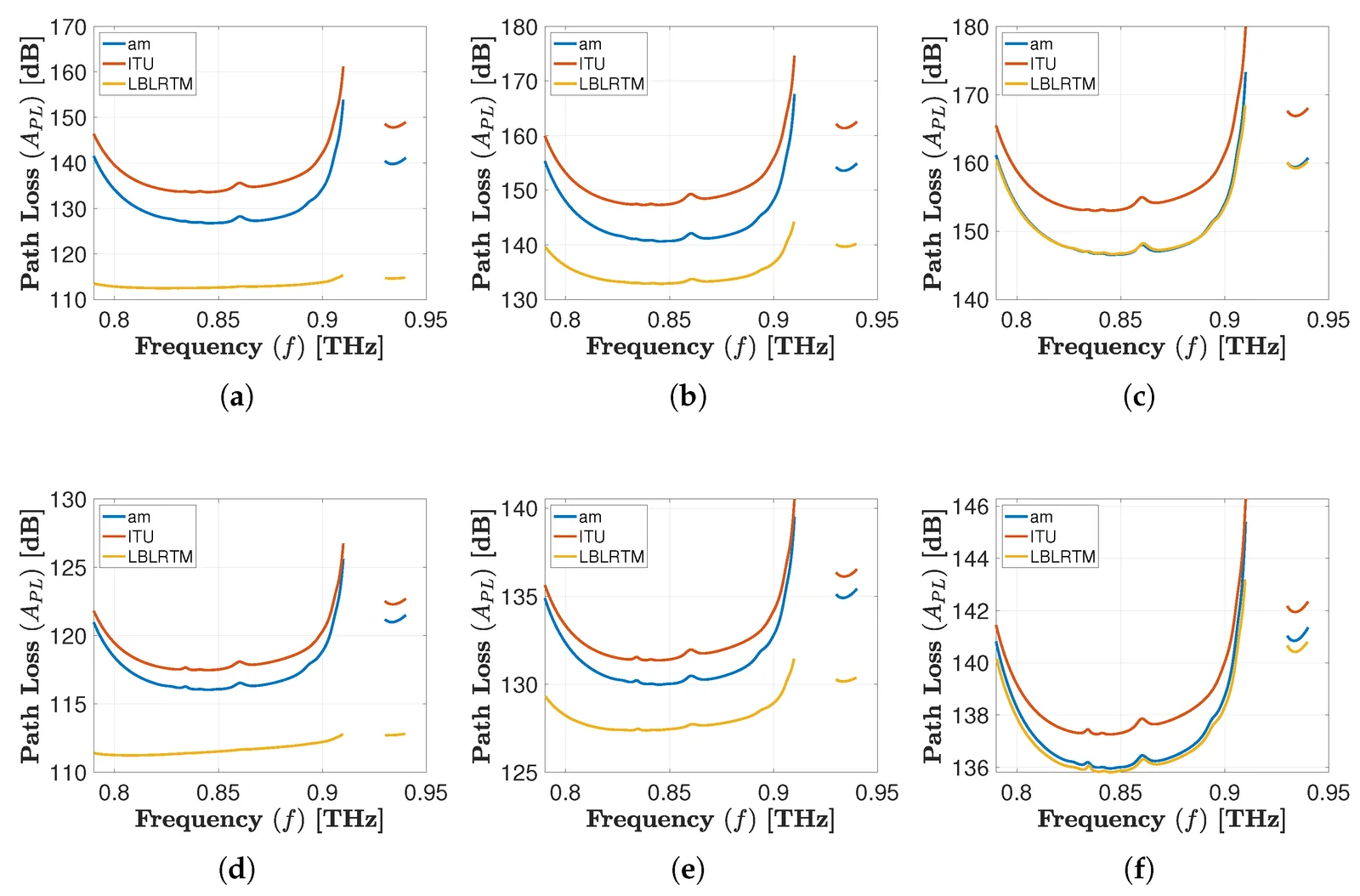
Path Loss in dB for all three tools, for vertical communication at various distances with h=100 m, where the first drone is at altitude $h_{1}=h$ m, and the second drone is at altitude $h_{2}=h+d_{v}$ m (Only data points inside Band 1 (0.79–0.91 THz) and Band 2 (0.93–0.94 THz) are plotted, appearing discrete). (**a**) Tropical, at $d_{v}=10$ m. (**b**) Tropical, at $d_{v}=50$ m. (**c**) Tropical, at $d_{v}=100$ m. (**d**) U.S. Standard 1976, at $d_{v}=10$ m. (**e**) U.S. Standard 1976, at $d_{v}=50$ m. (**f**) U.S. Standard 1976, at $d_{v}=100$ m.

In Figure 6, total path loss with respect to frequency is shown, where the first drone is at an altitude, h=100 m, and the second drone is $d_{v}$ meters away, communicating vertically, for three tools. The first row, with Figure 6a–c shows total path loss under a tropical weather profile, and the second row shows the U.S. Standard 1976 for three tools, with Figure 6d–f. As in the horizontal scenarios, the path loss is higher in the tropical weather profile because of the higher water vapor concentration. Furthermore, as seen in the figure, the path loss difference among the tools is higher in the vertical scenario than in the horizontal scenario.

**Figure 7:**
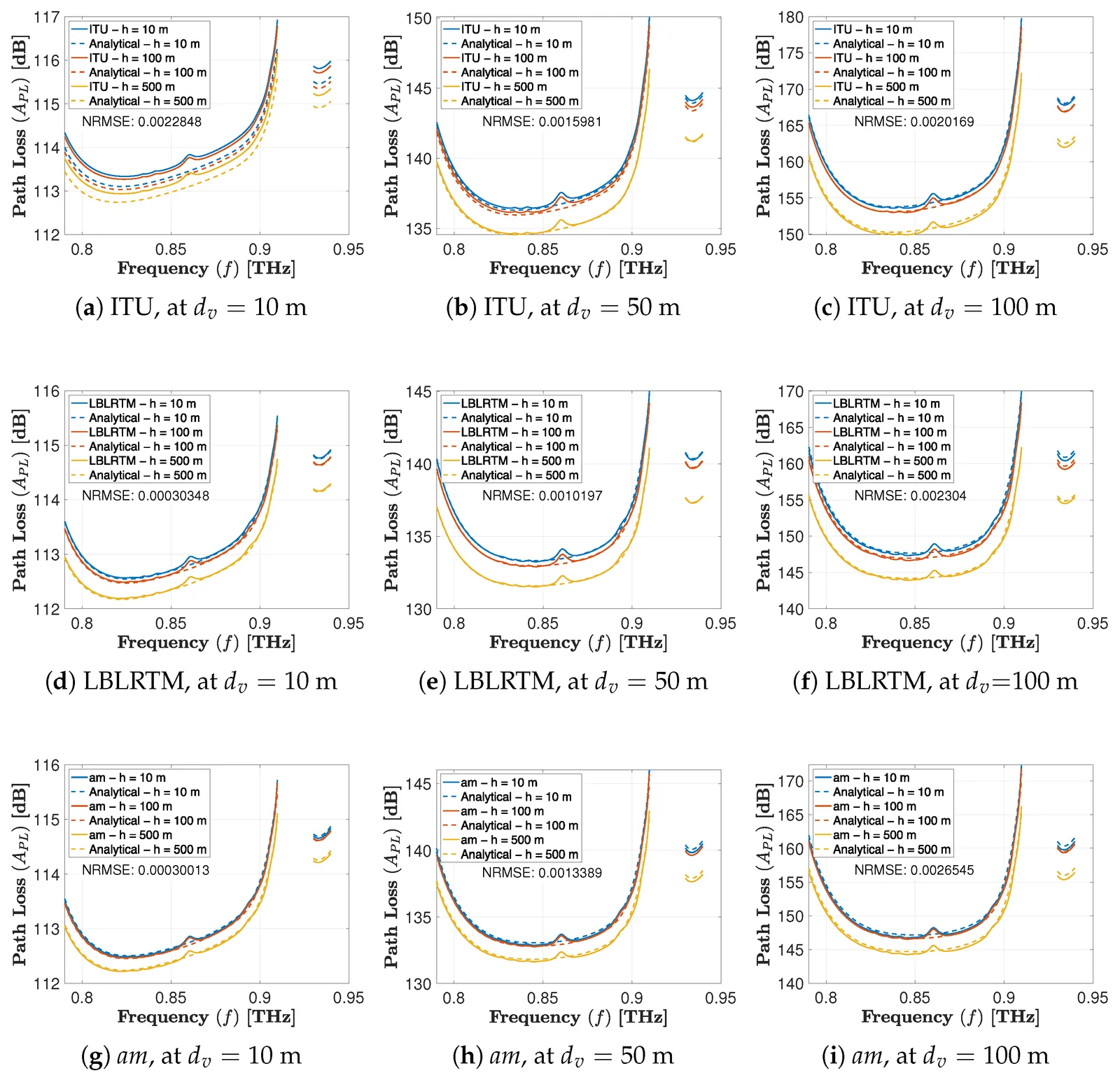
Path Loss in dB and the simple analytical model (dashed lines) [11] for ITU, LBLRTM and *am* under a tropical weather profile, for vertical communication at various distances and h=10,100,500 m, where the first drone is at altitude $h_{1}=h$ m, and the second drone is at altitude $h_{2}=h+d_{v}$ m (Only data points inside Band 1 (0.79–0.91 THz) and Band 2 (0.93–0.94 THz) are plotted, appearing discrete).

**Figure 8:**
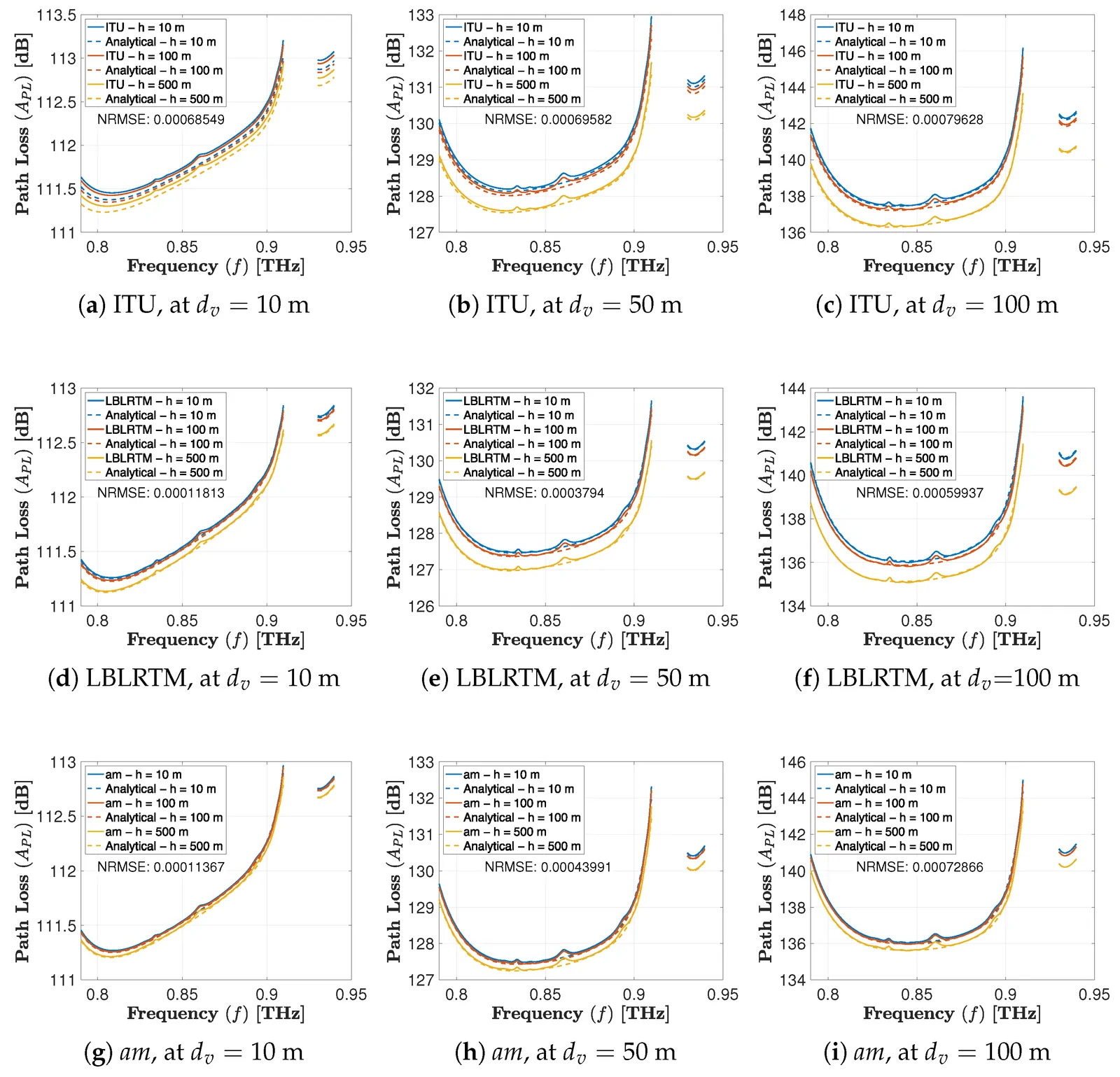
Path Loss in dB and the simple analytical model (dashed lines) [11] for ITU, LBLRTM and *am* under the U.S. Standard 1976 profile, for vertical communication at various distances and h=10,100,500 m, where the first drone is at altitude $h_{1}=h$ m, and the second drone is at altitude $h_{2}=h+d_{v}$ m (Only data points inside Band 1 (0.79–0.91 THz) and Band 2 (0.93–0.94 THz) are plotted, appearing discrete).

Fitting of the simple analytical model [11] is carried out in the same manner as the horizontal scenarios in the previous subsection, for the same two bands. Figure 7 displays the total path loss and the simple analytical model fitted on each tool’s data and scenario separately under a tropical weather profile. In each subfigure, the first drone is at h=10,100,500 m, and the second drone is $d_{v}$ meters apart, communicating vertically. Similarly, Figure 8 shows the same setups under the U.S. Standard 1976 weather profile. The NRMSE is also given in each subfigure for the data and prediction points in that subfigure.

### 4.4. Analytical Model Results

The fitting is performed utilizing the simple analytical model [11] with the same hyperparameter values and different coefficient values specific to the data obtained from a tool for various scenarios, weather profiles, distances, and altitudes. The NMRSE values are calculated as in (7) by inserting all path loss data points generated by an absorption modeling tool and all corresponding predicted values obtained from the analytical model.

Table 4 provides the NRMSE values for the considered tools and scenarios for both weather profiles. The results indicate that the lower NRMSE values are observed in horizontal communication. This outcome aligns with the earlier observation, which suggests that in horizontal communication scenarios, weather composition remains relatively consistent with increasing distance, given that weather profiles are modeled based on altitude. On the other hand, vertical communication scenarios involve signal propagation through multiple atmospheric layers, where temperature, pressure, and water vapor concentration vary with altitude, showing higher variability in weather composition as distance increases, likely contributing to the less accurate fit of the simple analytical model in vertical scenarios.

**Table 4:** NRMSE for different tools and scenarios.

	U.S. Standard Horizontal	U.S. Standard Vertical	Tropical Horizontal	Tropical Vertical
**LBLRTM**	$3.48\;\times \;10^{-4}$	$3.98\;\times \;10^{-4}$	$1.08\;\times \;10^{-3}$	$1.29\;\times \;10^{-3}$
**ITU**	$3.48\;\times \;10^{-4}$	$7.80\;\times \;10^{-4}$	$1.16\;\times \;10^{-3}$	$2.04\;\times \;10^{-3}$
***am***	$4.05\;\times \;10^{-4}$	$4.34\;\times \;10^{-4}$	$1.28\;\times \;10^{-3}$	$1.55\;\times \;10^{-3}$

Moreover, when comparing the NRMSE values for the U.S. Standard 1976 and the tropical weather profile in horizontal scenarios, the differences are negligible, suggesting relatively similar model performance across these profiles. However, in the vertical scenarios, the NRMSE is significantly higher for the tropical weather profile. This difference might be attributed to the higher water vapor concentration, which could lead to more pronounced effects of highly varying frequency-selective attenuation windows, particularly in the vertical communication scenario. This challenges the simple analytical model the most under the tropical and vertical scenario.

The consistency of the tools is supported both by the similarity in their absorption loss values and the low NRMSE values. As shown in Table 4, all the tools achieve NRMSE values on the order of $10^{-3}$ or lower in horizontal scenarios, indicating a strong agreement. Additionally, the LBLRTM and *am* tools produce very close results, while the ITU model shows minor deviations, especially in vertical scenarios. Nonetheless, the absorption loss predictions across all tools remain within comparable and acceptable ranges, validating the robustness of the models used.

It is not reasonable to label the tools as accurate or inaccurate based on the NRMSE values, as these values do not reflect errors from the experimental results but rather a measure of the fit of the simple analytical model to different scenarios. Among the three tools, the lowest NRMSE is observed with LBLRTM, likely because the simple analytical model was initially developed based on data generated by LBLRTM under a horizontal scenario in [11], also tuning the hyperparameters for that specific case. Even if that is the case, the analytical model achieves a sufficiently good fit, and these results validate the model and generalize the results of [11] for broader applicability for the simple analytical model for these frequency bands.

## 5. Conclusions

In this paper, we provide a comprehensive comparison of molecular absorption loss modeling tools, namely ITU, LBLRTM, *am*, and HotW, specifically for THz band drone-to-drone and drone-to-ground communications, using horizontal and vertical scenarios, considering the tropical and U.S. Standard 1976 weather profiles. In addition to data generation and path loss simulation comparisons for drone altitudes at various distances, the simple analytical model is used to test the data generated by the tools.

As reflected in figures and Table 4, the simple analytical model achieves the lowest fitting error when applied to data from the LBLRTM tool and under horizontal communication scenarios. The model also achieves good fitting performance for ITU and *am* for horizontal scenarios. The increased variability in the weather profiles in the vertical scenarios makes it more challenging to accurately capture molecular absorption loss using a simplified model. This leads to higher normalized fitting errors in vertical cases, especially since the model was originally designed using LBLRTM data under horizontal scenarios. However, the overall consistency of the tools is supported by their closely matching absorption loss values and the low NRMSE values observed in horizontal scenarios.

The comparison of these THz band molecular absorption loss modeling tools demonstrates that, despite having differences in design and capabilities, a simulation tool may be selected based on the specific application and requirements since they produce similar attenuation values. This detailed comparison of molecular absorption loss modeling tools offers valuable insights about THz band drone communication since it is the first study that compares these tools across a variety of drone communication scenarios and weather profiles.

With the increase in THz band experimental studies, more measurement data can be obtained under specific setups, including drone scenarios. As such results become available, as future work, the considered THz modeling tools can be evaluated regarding their ability to calculate molecular absorption loss by comparing their data with experimental results. Furthermore, comparisons can be extended by considering other atmospheric conditions, such as rain and fog.

## Figures and Tables

**Table 2:** Tropical weather profile data for various altitudes [3,20].

Altitude (*h*) (m)	Temperature (*T*) (^o^C)	Pressure ( ρ ) (mbar)	Water Vapor Density (*V*) (g/m^3^)	Water Vapor Density (*V*) (ppm)
108	24.75	1000	17.3249	$2.44\times 10^{4}$
328	22.95	975	16.4504	$2.36\times 10^{4}$
554	21.55	950	15.3036	$2.24\times 10^{4}$
785	20.15	925	13.9899	$2.09\times 10^{4}$
1021	18.95	900	12.5126	$1.91\times 10^{4}$
1263	19.05	875	11.0093	$1.72\times 10^{4}$

**Table 3:** U.S. Standard 1976 Weather Profile Data for various altitudes [32,36,37].

Altitude (*h*) (m)	Temperature (*T*) (^o^C)	Pressure ( ρ ) (mbar)	Water Vapor Density (*V*) (g/m^3^)	Water Vapor Density (*V*) (vmr)
0	15.000	1013.250	5.857	$7.750\times 10^{-3}$
1000	8.501	898.762	4.171	$6.070\times 10^{-3}$
2000	2.004	795.014	2.885	$4.630\times 10^{-3}$

## Data Availability

The original contributions presented in this study are included in the article. These data were derived from the following resources available in the public domain: [23,29,33,34]. Further inquiries can be directed to the corresponding author.

## Abbreviations

The following abbreviations are used in this manuscript:

5G	Fifth-Generation
6G	Sixth-Generation
AER	Atmospheric and Environmental Research
*am*	Atmospheric Model
CFA	Center for Astrophysics
EM	Electromagnetic
FSPL	Free Space Path Loss
HITRAN	High-Resolution Transmission Molecular Absorption Database
HotW	HITRAN on the Web
ITU	International Telecommunication Union
LBLRTM	Line-by-Line Radiative Transfer Model
MERRA-2	Modern-Era Retrospective Analysis for Research and Applications, Version 2
NASA	National Aeronautics and Space Administration
NRMSE	Normalized Root Mean Square Error
NTN	Non-Terrestrial Network
RMSE	Root Mean Square Error
THz	Terahertz
XR	Extended Reality
