# Correction: Sekeroglu et al. Absorption Loss Modeling Tools for Terahertz Band Drone Communications. *Sensors* 2025, *25*, 4957

**DOI:** 10.3390/s26165141

**Published:** 2026-08-14

**Authors:** Berkay Sekeroglu, Mikail Erdem, Ozgur Gurbuz, Akhtar Saeed, Hayrettin Cagan Sendag, Murat Kulaksizoglu

**Affiliations:** 1 Department of Electrical and Electronics Engineering, Bogazici University, Istanbul 34342, Türkiye; 2 Faculty of Engineering and Natural Sciences, Sabanci University, Istanbul 34956, Türkiye

## Table Correction

A data-loading error in one of the scripts affected the numerical fitting results for the ITU Model and the am Tool presented in Table 4 in the original publication [1]. The correct version of Table 4 appears below. The authors state that the scientific conclusions are unaffected. This correction was approved by the Academic Editor. The original publication has also been updated.

## In-Line Equation Correction

In the original publication [1], there was an error in the in-line equation about analytical total path loss prediction in the sixth paragraph of Section 4.2. The correct sentence is given below.

The total path loss modeled by the fitted simple analytical model is denoted as ${\hat{A}}_{PL}\left(d,f,h\right)=10\log_{10}\left(1/\hat{\tau }\left(d,f,h\right)\right)+20\log_{10}\left(\{4\pi df\}/c\right)$.

The authors state that the scientific conclusions are unaffected. This correction was approved by the Academic Editor. The original publication has also been updated.

## Figures and Tables

**Table 4:** NRMSE for different tools and scenarios.

	U.S. Standard Horizontal	U.S. Standard Vertical	Tropical Horizontal	Tropical Vertical
**LBLRTM**	$3.48\;\times \;10^{-4}$	$3.98\;\times \;10^{-4}$	$1.08\;\times \;10^{-3}$	$1.29\;\times \;10^{-3}$
**ITU**	$3.48\;\times \;10^{-4}$	$7.80\;\times \;10^{-4}$	$1.16\;\times \;10^{-3}$	$2.04\;\times \;10^{-3}$
***am***	$4.05\;\times \;10^{-4}$	$4.34\;\times \;10^{-4}$	$1.28\;\times \;10^{-3}$	$1.55\;\times \;10^{-3}$
